# Stimulation of the Angular Gyrus Improves the Level of Consciousness

**DOI:** 10.3390/brainsci9050103

**Published:** 2019-05-06

**Authors:** Liudmila Legostaeva, Alexandra Poydasheva, Elizaveta Iazeva, Dmitry Sinitsyn, Dmitry Sergeev, Ilya Bakulin, Dmitry Lagoda, Elena Kremneva, Sofya Morozova, Yulia Ryabinkina, Natalia Suponeva, Michael Piradov

**Affiliations:** Research Center of Neurology, Volokolamskoe shosse, 80, Moscow 125367, Russia; alexandra.poydasheva@gmail.com (A.P.); lizaveta.mochalova@gmail.com (E.I.); d_sinitsyn@mail.ru (D.S.); dmsergeev@yandex.ru (D.S.); bakulinilya@gmail.com (I.B.); dmitrylagoda.doc@gmail.com (D.L.); Moomin10j@mail.ru (E.K.); kulikovasn@gmail.com (S.M.); Ryabinkina11@mail.ru (Y.R.); nasu2709@mail.ru (N.S.); mpi711@gmail.com (M.P.)

**Keywords:** disorders of consciousness, angular gyrus, repetitive transcranial magnetic stimulation, neuromodulation, vegetative state, non-invasive brain stimulation

## Abstract

Background: Navigated repetitive transcranial magnetic stimulation (rTMS) is a promising tool for neuromodulation. In previous studies it has been shown that the activity of the default mode network (DMN) areas, particularly of its key region—the angular gyrus—is positively correlated with the level of consciousness. Our study aimed to explore the effect of rTMS of the angular gyrus as a new approach for disorders of consciousness (DOC) treatment; Methods: A 10-session 2-week high-frequency rTMS protocol was delivered over the left angular gyrus in 38 DOC patients with repeated neurobehavioral assessments obtained at baseline and in 2 days after the stimulation course was complete; Results: 20 Hz-rTMS over left angular gyrus improved the coma recovery scale revised (CRS-R) total score in minimally conscious state (MCS) patients. We observed no effects in vegetative state (VS) patients; and Conclusions: The left angular gyrus is likely to be effective target for rTMS in patients with present signs of consciousness.

## 1. Introduction

Some patients who have survived prolonged coma after severe brain damage and regained wakefulness develop disorders of consciousness (DOC). Depending on the presence of observable signs of consciousness, these states can be divided into the vegetative state (VS, also called the unresponsiveness wakefulness syndrome, UWS) and the minimally conscious state (MCS). While MCS patients have minimal but persistent reproducible behavioral evidence of self-awareness and reactivity, VS patients completely lack any apparent signs of cognitive activity.

Chronic DOC is clinically challenging as prognosis for functional recovery is poor for the majority of patients, and effective pharmacologic therapy is currently lacking [1,2]. Neurostimulation techniques have been recognized as promising experimental approaches to DOC treatment. Nowadays non-invasive brain stimulation methods (NIBS), such as transcranial direct current stimulation (tDCS) and repetitive transcranial magnetic stimulation (rTMS), prove themselves as promising tools. Few studies have addressed the application of NIBS, with conflicting results [3]. Thibaut et al. in 2014 observed transiently improved signs of consciousness in 13 of 30 MCS patients following severe brain damage as measured by changes in the CRS-R total scores after a single session of tDCS over the left dorsolateral prefrontal cortex (DLPFC), while no effect was seen in VS/UWS subjects [4]. Similar results were obtained in another study using a five-day course of anodal tDCS in MCS patients [5]. Finally, the recently reported results of a study of prolonged home-based tDCS also demonstrated an improvement of signs of consciousness in MCS patients [6]. Stimulation of the right DLPFC with rTMS resulted in some behavioral gain in one posttraumatic VS patient and transient improvement in three of 10 post-anoxic UWS patients; improvement in the levels of consciousness and behavior was also reported by Xie et al. in a set of UWS, MCS and comatose patients [7,8]. When applied over the left DLPFC, rTMS resulted in a notable clinical improvement in MCS subjects in contrast to VS patients in a study by Xia et al. [9]. Piccione et al. in 2011 reported that 20Hz rTMS delivered on the left primary motor cortex (M1) had improved awareness and arousal in an MCS patient [10]. On the other hand, no effects of rTMS of the motor cortex in VS patients were reported by a randomized, sham-controlled study; similar findings were obtained in a set of VS and MCS patients by Liu et al. [9,11]. Protocols of stimulation in the abovementioned studies varied widely which also may be the reason for inconclusive results. Overall, the results of NIBS studies suggest that neuromodulation of certain regions involved in the networks supporting consciousness is worth further investigation as a safe and potentially effective approach for facilitating recovery in chronic DOC patients.

A key question is the choice of a brain area may potentially produce clinically significant behavioral improvement in DOC patients and is suitable for non-invasive stimulation. The structural architecture and functional interactions supporting consciousness are poorly understood, however, there are evidences that functional and structural connectivity within the default mode network (DMN) correlate with the level of behavioral responsiveness in DOC patients [12,13,14]. Perhaps due to the close connection of DMN regions to introspective cognition and memory—visual imagery is linked to activation of supramodal and frontoparietal areas associated with attention and cognitive control and visual cortical regions most strongly activated by visual perception itself and [15,16,17]. Furthermore, connectivity within DMN may have prognostic value for the recovery of consciousness [18,19]. Several areas of DMN, namely posterior cingulate cortex and precuneus, may be the subject of special attention as intrinsic connectivity within these brain areas significantly correlated with consciousness level and recovery [18]. The decreases of connectivity and metabolism in these regions of the DMN are observed as a key marker of the impairment of consciousness [12,13,20,21,22,23,24,25,26,27].

Based on these studies we aimed to choose those DMN regions, stimulation of which may potentially affect consciousness-related brain networks and that are technically feasible for transcranial magnetic stimulation. We particularly suggest left angular gyrus as a target for TMS due to its significant relations to other regions of the brain involved in a number of processes such as language, number processing and spatial cognition, memory retrieval and attention (Brodmann area 39). The angular gyrus occupies a central location from a neuroanatomical perspective to act as a multimodal convergence hub within the DMN. It receives inputs from the primary sensory cortices and integrates sensory information of different modalities [28,29,30]. These mechanisms may extend the role of the angular gyrus into post-sensory processes such as memory retrieval [31,32]. All these facts demonstrate the multimodal integrative functions of the angular gyrus and reflect its possible role in the process of consciousness. In contrast to the cingulate cortex, the angular gyrus is located on the convexital surface of the brain and is easily accessible for transcranial stimulation.

Therefore, we aimed to verify whether repeated sessions of rTMS navigated to the left angular gyrus may produce clinical behavioral improvements (assessed by CRS-R) in DOC patients.

## 2. Materials and Methods

We developed an open non-randomized study protocol that was approved by the local ethics committee of the Research Center of Neurology. All patients’ legal representatives signed an informed consent form approved by the local ethics committee before enrollment.

### 2.1. Eligibility Criteria and Clinical Assessment

**Figure 1:**
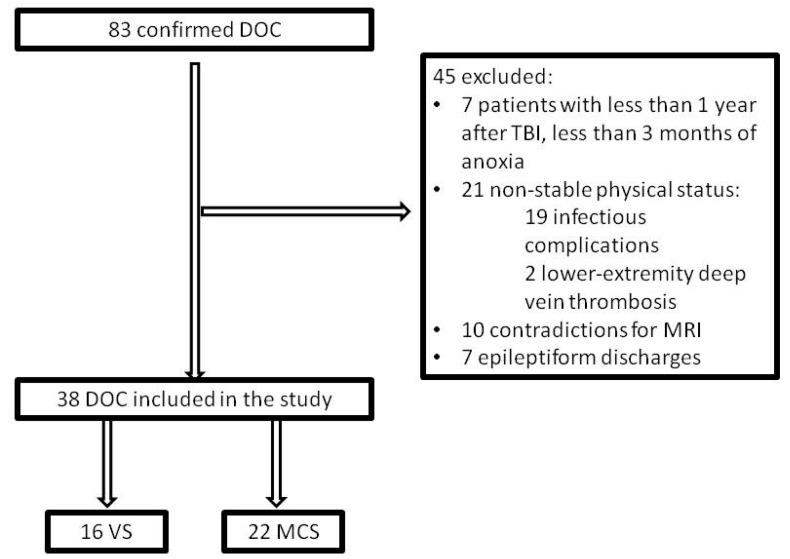
Study flow diagram. DOC: disorders of consciousness; TBI: traumatic brain injury; VS: vegetative state; MCS: minimally conscious state.

Inclusion criteria were a history of traumatic or anoxic brain injury and verified DOC status. Participants were excluded if they were in non-stable clinical status (e.g., acute myocardial infarction, deep vein thrombosis, or episodes of pulmonary embolism, acute infections, sepsis, severe anemia, etc.) or had implanted medical devices such as cerebral shunt, cardiac pacemaker, intracardiac catheter, or electronic pump, metal plates closing skull defects, metallic staples or vascular sutures. Patients who showed epileptiform discharges on EEG screening were also excluded due to safety issues (see Figure 1).

Clinical assessment was performed by trained and experienced clinicians using the validated Russian version of the CRS-R score [33]. We applied internationally established criteria for diagnosing VS and MCS. CRS-R assessment was performed at baseline (before the stimulation) and in two days after the stimulation course was completed [34,35,36]. Changes from baseline in the total score of the CRS-R and in the scores of the six CRS-R subscales addressing auditory, visual, motor, oromotor/verbal, communication, and arousal processes were tested with the Wilcoxon signed-rank test. Statistical analyses were performed using Matlab (2017a, MathWorks, Natick, MA, USA).

### 2.2. Structural MRI

**Figure 2:**
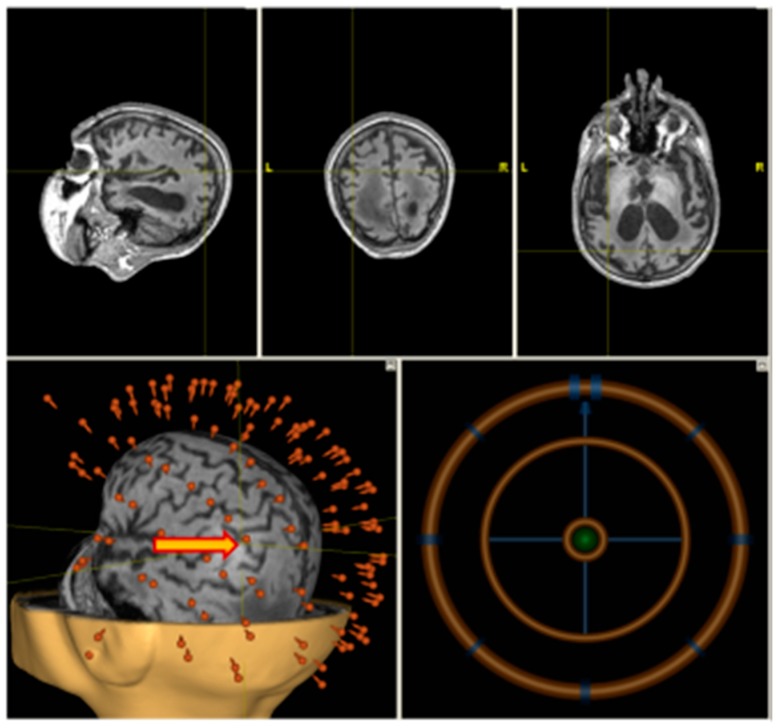
Individual navigation (Nexstim) on left angular gyrus.

A high-resolution T1-weighted anatomical scan (MP–RAGE or SPGR) was obtained using Siemens MAGNETOM Verio 3T (Siemens AG, Muenchen, Germany) clinical scanner. A total of 176 sagittal slices were acquired to cover the whole brain. Anatomical imaging was co-registered with a patient’s head in the neuronavigation system (Nexstim Ltd., Helsinki, Finland). The stimulation point corresponding to the anatomical location over the angular gyrus was chosen individually using the T1 scan (Figure 2).

### 2.3. rTMS

High frequency rTMS was delivered on a non-navigated Neurosoft stimulator (Neurosoft ltd., Ivanovo, Russia) (see Figure 2) with figure-eight coil.

We used the target transfer method allowing us to mark the desired sites of stimulation using the neuronavigated system to continue the therapeutic protocol on a non-navigated system in the intensive care unit to avoid transportation difficulties, and the stimulation point was marked on an individual skull-cap. All patients received an rTMS course consisting of 10 sessions, scheduled five times a week over two consecutive weeks. Stimulation intensity was determined individually as 80% of the resting motor threshold (RMT). RMT was defined as the lowest stimulation intensity which produced a visible hand muscle twitch in five out of 10 trials. In four patients RMT estimation was impossible since no twitch was evoked even with the maximum stimulator intensity. In these patients 40% of the maximum stimulator intensity was used. Each rTMS session consisted of 3200 stimuli applied with 20 Hz frequency (stimulation train duration—4 s, inter-train interval—26 s) for 20 minutes.

During the rehabilitation all patients received a course of 10 physical therapy sessions, each lasting 45–55 minutes, as well as robotic verticalization, scheduled five times a week for two consecutive weeks. The physical therapy focused on passive joint movements and the prevention of contractures with consideration of each patient’s individual limitations.

### 2.4. Statistical Analysis

The Wilcoxon signed-rank test was used for testing the effect of the therapy. We employed nonparametric methods because they do not assume normality of the data, and the distribution of CRS-R score changes between sessions was neither known from the literature nor could be reliably derived using the present data, because distribution tests typically require considerably larger samples for good power [37]. The test was applied to the CRS-R total score as a comprehensive measure of behavioral signs of consciousness.

## 3. Results

Eighty-three patients with confirmed DOC diagnosis were screened for the study at the Research Center of Neurology (Moscow, Russia). To minimize the probability of confounding effect by spontaneous recovery, we excluded anoxic patients less than three months post-incident and traumatic brain injury (TBI) patients at less than 12 months since insult (seven patients excluded in total). Twenty-one patients were excluded because of their unstable clinical status due to severe anemia, acute myocardial infarction, episodes of pulmonary embolism, vein thrombosis, infections complications, decubitus ulcers, etc. Ten patients had contraindications to MRI, such as implanted medical devices, e.g., cerebral shunt, cardiostimulator, intracardiac catheter, electronic pump, or metal plates closing skull defects, metallic staples or vascular sutures. The remaining 45 patients underwent EEG monitoring, which identified epileptiform signs in seven patients, who were excluded.

We assigned thirty-eight patients (16 women, mean age 36.42, range 18–67 years) to receive rTMS delivered over the left angular gyrus. The group demographic data is performed in Table 1.

All patients were divided into two subgroups according to their DOC status (VS or MCS). VS patients (*n* = 16) had a mean age of 36.25 (min: 19; max: 59); seven were women; 15 were post-anoxic, mean interval since awakening after coma was 1 year 7 months (min 3 months, max 3 years and 3 months), and 1 was traumatic (interval since emergence from coma was 2 years and 3 months). MCS patients (*n* = 22) had a mean age 36.54 (min: 18; max: 67); nine of them were women; proportions of anoxic and post-traumatic patients were equal (11/11). Mean interval since arousal after coma in group with anoxia was 1 year and 4 months (min 3 months; max 2 years and 6 months) and in traumatic patients—1 year 9 months (min 1 year; max 3 years and 1 month). There were no significant differences in age between VS and MCS groups (*p* = 1, Mann–Whitney test. The individual findings are summarized in Table 2.

All patients completed the course of rTMS. No adverse events related to stimulation were reported.

The behavioral improvement after the rTMS course was assessed by means of the validated Russian version of the CRS-R scale. The baseline mean score was 5 (min: 4; max: 7) in VS and 14 (min: 7; max: 21) in MCS (see Table 2). Subclinical changes are surely fluctuating in MCS patients, but the use of a standardized clinical approach such as CRS-R scale reduces the possibility of establishing an incorrect diagnosis.

**Figure 3:**
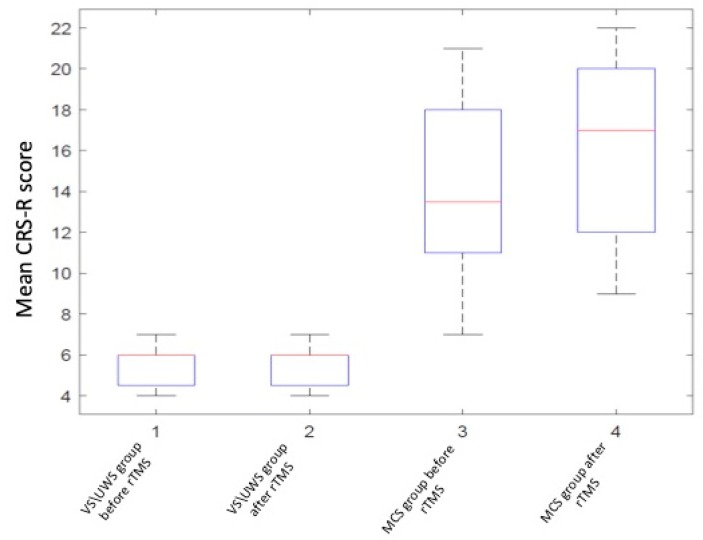
Coma recovery scale revised (CRS-R) score changes in group analysis.

**Figure 4:**
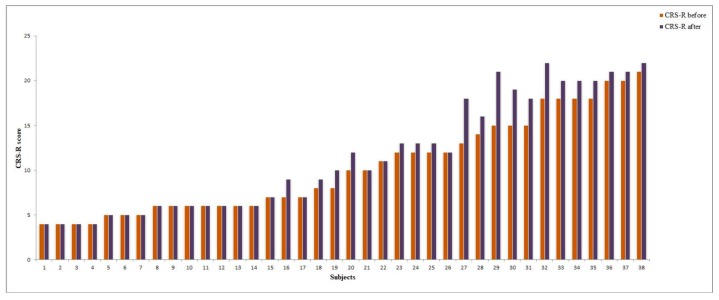
CRS-R score change after rTMS in vegetative state (VS) and minimally conscious state (MCS) patients (individual data).

The CRS-R total score significantly increased after stimulation in the MCS subgroup (*p* = 0.0001, two-sided Wilcoxon signed-rank test); in the VS subgroup no significant differences were found (*p* > 0.05) (see Table 2, Figure 3 and Figure 4).

**Figure 5:**
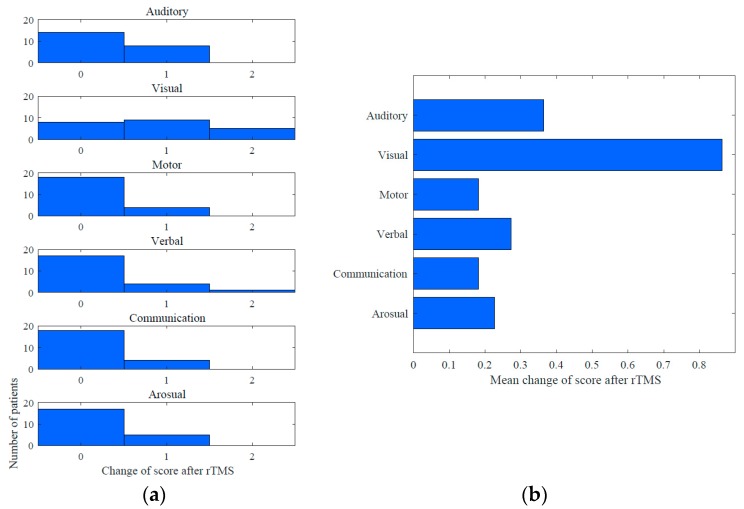
Mean changes in CRS-R score subscales in MCS group: (**a**) mean change into CRS-R subscales after rTMS course; (**b**) improvements were observed in subscores.

Improvements in the CRS-R score after the rTMS course were observed in 19/22 (86.3%) MCS patients (Figure 4). The mean increase in the CRS-R total score was 2,1. Among the CRS-R subscales, the largest improvements were observed in the visual (mean change 0.86), auditory (0.36) and verbal (0.27) scores (see Figure 5).

In three traumatic MCS patients, object recognition was observed after rTMS, whereas at baseline they had only shown visual pursuit. In two anoxic MCS patients the visual function changed from fixation to object reaching after the stimulation course. In one case of MCS there was a dramatic improvement in verbal signs. The patient progressed from oral reflexive movement to articulation with yes/no answers, attempts to pronounce her name, etc. This case represented how the subclinical changes improved the DOC diagnosis quality to emergence from minimally conscious state (EMCS) by means of CRS-R scoring. In other cases of MCS the diagnosis remain the same despite positive CRS-R changes. No significant effect of etiology on CRS-R improvement within the MCS subgroup was found (*p* = 0.3, two-sided Mann–Whitney test). Figure 5 shows the CRS-R subscale score changes for MCS patients.

## 4. Discussion

The present study is the first application of 20 Hz-rTMS over the left angular gyrus at 80% of the individual RMT for 10 sessions in DOC patients. Each session lasted 20 minutes and did not cause any side-effects. We observed significant improvement in the CRS-R total score in the MCS subgroup, whereas in the VS/UWS subgroup there was no significant effect.

As there are no pharmacological treatments that provide clear evidence on improvement of restoration of consciousness in chronic DOC patients, alternative strategies of rehabilitation, that involve external modulation of cortico-cortical and corticothalamic neural loops, increasingly attract attention. Currently, several techniques of stimulation are proposed, including non-invasive (tDCS and rTMS) and invasive (DBS) approaches, both showing some effectiveness in the recovery of consciousness [28]. In this protocol technically feasible non-invasive rTMS approach was selected. rTMS is known to depolarize neurons under the stimulating coil and indirectly affect on areas related to cognition and behavior and its application in the high-frequency protocols may induce an increased release of dopamine which may modulate the neuronal activity [38].

Positive effects of rTMS over DLPFC or M1 area in chronic DOC setting were seen in several studies, while others did not demonstrate significant improvement [7,9,10,11,39,40,41]. However, not only the site of stimulation but the frequency and exposure to rTMS varied across these studies, and currently there is no unified protocol for rTMS in DOC patients. Based on the aforementioned results, we decided to apply protocol with high-frequency stimulation aimed at the restoration of impaired neuronal connectivity within DMN pathways [42]. Seizures are often seen in patients with VS/UWS or MCS, and to ensure safety of the high-frequency stimulation we excluded patients with epileptic discharges revealed at screening EEG [43,44]. Previous studies showed that effects of a single session of rTMS varied and even if the effect is present, it is transient [10,39,41]. Thus a single session may not be enough to provide a significant effect. As prolonged exposure to rTMS is assumed to increase its possible efficacy, our protocol included repeated stimulation for a total of 10 sessions.

The choice of the target for stimulation was based on recent studies that explored the role of the left angular gyrus in the framework of neural correlates of consciousness. From a neuroanatomical perspective, this area lies at the confluence of brain regions supporting episodic memory, language, attentional, semantic, numerical, and social cognitive processes [28,29]. It also receives visual, auditory, and visuomotor input from primary sensory cortices and sensory association areas [28,30,45]. Due to its location, the angular gyrus has a role of a multimodal convergence hub. It is placed to integrate incoming sensory and cognitive information to create unified representations [29,46]. Moreover, being a node of the default mode network it also connects with the frontoparietal control network, which is implicated into executive control during cognitive processing [47]. Activation in the angular gyrus remains blind to the modality of the recollected content and the type of episodic retrieval task, which indicates its multimodal processing role [48,49]. The angular gyrus is also connected with the precuneus and the mid-cingulate cortex. This area was proposed to mediate different aspects of memory function, including memory retrieval tasks [50]. It also responds to familiarity of stimuli during learning and memory as a part of the “parietal memory network” [50,51,52]. Moreover, in a meta-analysis of the attention and memory systems, it was stated that the inferior parietal cortex, which includes the supramarginal gyrus and the angular gyrus, is part of an attentional subsystem that mediates the automatic allocation of attention to task-relevant information, particularly in attending to retrieved memories [53,54]. One of our aims in the application of rTMS over the angular gyrus was to describe this area as the hub-modulator of the integration mechanism inside the process of consciousness. This state is supported by clinical heterogeneous changes in MCS patients group with the dramatic improvement in verbal and visual signs. In particular, the visual signs improvement can be also linked to imagery domain—the activation of visual regions are strongly associated with memory processing (hippocampus, parahippocampal cortex) and the posterior cingulate/precuneus (BA 31) that correlates with the vividness ratings of autobiographical memory [17,55,56].

Mild effect of rTMS in our study was seen only in MCS patients, which is in line with the studies of tDCS by Thibaut and rTMS over left DLPFC by Xia [9]. No improvement was observed in VS/UWS group, and this might be related to the poor capacity for neural plasticity in VS/UWS patients [57]. Of note, the majority of VS/UWS patients (15/1) in our study were of anoxic etiology, and clinical inefficacy of rTMS in this category of subjects may be related to a complete (or almost complete) derangement of cortical connectivity. This may have resulted in the absence of neural networks capable to act as an efficient substrate for the long-term effects of rTMS. However, at present there is no evidence that other cortical targets of stimulation may produce behavioral effects in VS/UWS patients with both non-traumatic and traumatic etiologies, neither using rTMS nor tDCS [4,7,11,39,40]. In the MCS group there was no significant difference in the results between the traumatic and non-traumatic subgroups. Thus, we assume that the heterogeneous etiology of our VS/UWS group did not affect the results of the stimulation. However, the question of the possible dependence of the rTMS effect on the etiology needs further study.

MCS patients seem to react behaviorally to the rTMS of the angular gyrus, and the largest function improvements were observed in the visual, auditory and verbal behavioral measures. This may be explained by a spread of the modulation effect from the angular gyrus to all the areas connected to it. We suggest that the absence of effect in three MCS patients might be associated with partial disconnections and lesions of the brain. In the case of the EMCS patient the dramatic subclinical improvement might be spontaneous but due to the timing of impaired consciousness is rather improbable.

Thus, rTMS may also be useful in identifying subgroups of MCS patients who could benefit from more invasive and potentially more effective stimulation strategies such as DBS. Cortical plasticity and changes in connectivity are central to the recovery of consciousness in DOC patients. We assume that neuromodulation, such as NIBS, applied to an area within a given neural network (e.g., the angular gyrus in the DMN) can lead to the activation of the whole network. Our hypothesis was supported by plenty of studies using fMRI and PET connectivity in DOC patients [12,20,24]. It has been shown that there is a significant difference in connectivity between DOC patients and healthy subjects. DOC patients have decreased glucose standardized uptake value in the thalamus, precuneus/posterior parietal cortex, inferior parietal, mesiofrontal cortex, as well as decreased DMN connectivity, compared with healthy subjects [14,19,58,59]. Graph theory also shows reduced modularity in VS/UWS and MCS compared with healthy subjects [60]. However, the contrast between the MCS and VS/UWS patients is more subtle and harder to pinpoint at the level of the overall connectivity. The DMN functional connectivity strength is able to differentiate MCS from VS/UWS patients [14]. Significant differences were found in the patterns of strong positive connections using node-specific whole-brain measures of connectome disruption [61].

We conclude that therapeutic brain stimulation techniques enhanced by neuroimaging-based target selection constitute a promising approach to the extremely challenging problem of DOC treatment.

There are some limitations in our study. The absence of a placebo group, which was due to the small number of patients eligible for the study, was partially compensated by selecting patients at sufficiently long intervals since anoxia/injury to avoid confounding by spontaneous recovery. However, in the future it is necessary to confirm the effect in controlled studies. Another limitation of the study is that the evaluation of the clinical effect was presented only once (2 days after the stimulation course), however it is planned to do a follow-up evaluation of the long-term effects.

Results added, in this study we employed only behavioral measure of rTMS effect, and future studies should include established instrumental approaches for the assessment of chronic DOC patients, such as EEG, TMS-EEG and fMRI that may promote further understanding of mechanisms underlying neuromodulation.

It is important to note, that in four cases the RMT could not be determined due to the absence of muscle twitches in response to TMS. We note that no adverse effects were observed in these patients. At the same time, the lack of data on the threshold could have led to an underestimation of the required simulation intensity, which may potentially have affected the effectiveness. Generally, it is also unclear how closely the motor threshold corresponds to the intensity of angular gyrus stimulation. Thus, there is a need for new methods of intensity selection in this group of patients (for example, based on TMS-EEG data).

## 5. Conclusions

This study is the first evaluation of the efficacy of rTMS applied over the left angular gyrus in disorders of consciousness. We obtained a clinical effect in MCS, but not in VS/UWS. These results provide a motivation for future research into the effectiveness of this new protocol. Additionally, the findings highlight the left angular gyrus as a promising target for therapeutic NIBS in DOC patients, which can help optimize the use of human and financial resources in DOC management.

## Figures and Tables

**Table 1:** Group demographic data.

	VS	MCS
*n*	16	22
age (median (upper quartile (UQ). lower quartile (LQ))	36 (19.59)	36 (18.67)
sex (Female/Male)	7/9	9/13
Etiology (anoxia/trauma)	15/1	11/11
Interval since anoxia/TBI months		
mean (min; max)	21 (3; 39)	20 (3; 38)
CRS-R score before rTMS		
mean (min; max)	5 (4; 7)	14 (7; 21)

VS: vegetative state; MCS: minimally conscious state; TBI: traumatic brain injury; rTMS: repetitive transcranial magnetic stimulation.

**Table 2:** Individual findings in coma recovery scale revised (CRS-R) before and after repetitive transcranial magnetic stimulation (rTMS) course.

ID	Sex	Age	Etiology	CRS-R before rTMS	CRS-R Subscales						CRS-R after rTMS	CRS-R Subscales					
					Auditory	Visual	Motor	Verbal	Com-n	Arousal		Auditory	Visual	Motor	Verbal	Com-n	Arousal
**UWS/VS1**	f	22	anoxia	4	0	0	1	1	0	2	4	0	0	1	1	0	2
**UWS/VS2**	f	27	anoxia	4	0	0	1	1	0	2	4	0	0	1	1	0	2
**UWS/VS3**	f	31	anoxia	7	1	1	2	1	0	2	7	1	1	2	1	0	2
**UWS/VS4**	f	47	anoxia	5	0	0	2	1	0	2	5	0	0	2	1	0	2
**UWS/VS5**	f	19	TBI	6	1	0	2	1	0	2	6	1	0	2	1	0	2
**UWS/VS6**	f	24	anoxia	7	2	0	2	1	0	2	7	2	0	2	1	0	2
**UWS/VS7**	f	47	anoxia	6	1	0	2	1	0	2	6	1	0	2	1	0	2
**UWS/VS8**	m	55	anoxia	4	0	1	1	1	0	1	4	0	1	1	1	0	1
**UWS/VS9**	m	21	anoxia	4	0	0	1	1	0	2	4	0	0	1	1	0	2
**UWS/VS10**	m	51	anoxia	5	0	1	1	1	0	2	5	0	1	1	1	0	2
**UWS/VS11**	m	22	anoxia	6	1	0	2	1	0	2	6	1	0	2	1	0	2
**UWS/VS12**	m	52	anoxia	5	1	0	1	1	0	2	5	1	0	1	1	0	2
**UWS/VS13**	m	47	anoxia	6	1	0	2	1	0	2	6	1	0	2	1	0	2
**UWS/VS14**	m	59	anoxia	6	1	0	2	1	0	2	6	1	0	2	1	0	2
**UWS/VS15**	m	31	anoxia	6	1	0	2	1	0	2	6	1	0	2	1	0	2
**UWS/VS16**	m	25	anoxia	6	1	0	2	1	0	2	6	1	0	2	1	0	2
**MCS1**	f	67	anoxia	15	3	5	3	1	1	2	18	4	5	4	2	1	2
**MCS2**	f	28	TBI	15	2	3	4	2	2	2	20	3	5	5	2	2	3
**MCS3**	f	56	anoxia	18	3	4	4	2	2	3	20	4	5	4	2	2	3
**MCS4**	f	25	TBI	7	1	1	2	1	0	2	9	1	3	2	1	0	2
**MCS5**	f	24	TBI	8	1	1	2	2	0	2	10	1	3	2	2	0	2
**MCS6**	m	53	anoxia	9	2	2	2	1	0	2	12	2	3	3	1	1	2
**MCS7**	m	44	TBI	14	2	2	3	2	2	3	16	3	3	3	2	2	3
**MCS8**	m	33	anoxia	12	1	2	3	2	1	3	13	2	2	3	2	1	3
**MCS9**	m	48	anoxia	10	2	1	3	1	1	2	12	2	3	3	1	1	2
**MCS10**	m	48	anoxia	9	2	2	2	1	0	2	10	2	3	2	1	0	2
**MCS11**	m	23	TBI	12	3	3	3	1	0	2	13	4	3	3	1	0	2
**MCS12**	m	47	anoxia	10	2	3	2	1	0	2	10	2	3	2	1	0	2
**MCS13**	m	18	TBI	11	2	3	3	1	0	2	11	2	3	3	1	0	2
**MCS14**	m	24	TBI	13	4	3	3	1	0	2	13	4	3	3	1	0	2
**MCS15**	f	32	anoxia	18	4	4	5	1	2	2	21	4	5	5	2	2	3
**MCS16**	f	24	TBI	20	4	5	5	1	2	3	22	4	5	6	2	2	3
**MCS17**	f	31	anoxia	18	4	4	5	1	2	2	20	4	5	5	1	2	3
**MCS18**	f	43	anoxia	13	3	3	3	1	1	2	19	4	4	3	3	2	3
**MCS19**	m	32	TBI	18	4	4	5	1	2	2	20	4	4	5	2	2	3
**MCS20**	m	55	anoxia	15	2	2	4	2	2	3	18	3	4	4	2	2	3
**MCS21**	m	20	TBI	20	4	5	5	2	1	3	21	4	5	5	3	1	3
**MCS22**	m	29	TBI	20	4	4	5	3	1	3	22	4	5	5	3	2	3

f, female; m, male.
